# Transpapillary/Transfistular Drainage for Two Refractory Postoperative Right‐Lobe Bilomas: A Case Report

**DOI:** 10.1002/deo2.70417

**Published:** 2026-09-11

**Authors:** Fumitaka Niiya, Naoki Tamai, Jun Noda, Tetsushi Azami, Yuichi Takano, Masatsugu Nagahama

**Affiliations:** ^1^ Division of Gastroenterology Department of Internal Medicine Showa Medical University Fujigaoka Hospital Kanagawa Japan

**Keywords:** biloma, endoscopic internalization, postoperative bile leak, transfistular drainage, transpapillary drainage

## Abstract

We report a case of two refractory postoperative right‐lobe bilomas that were successfully treated with transpapillary/transfistular (TP/TF) drainage. A 57‐year‐old man developed two bilomas (segments 6 and 7) after repeated hepatectomy for colorectal liver metastases. Initial endoscopic retrograde cholangiopancreatography with endoscopic nasobiliary drainage (ENBD) was performed; however, biloma enlargement was observed despite ENBD placement, prompting a switch to TP/TF drainage. Two 5‐Fr pigtail‐type ENBD tubes were placed within the biloma cavities, followed by internalization after confirmed biloma regression (B6: from 93 to 38 mm, B7: from 71 to 28 mm). As the leak improved from high‐grade to low‐grade, the drainage strategy was modified to plastic stent placement in the B7 bile duct, ultimately achieving complete biloma resolution approximately 8 months after TP/TF drainage. Stent‐free status has been maintained for 13 months, with a total follow‐up of 21 months. This case demonstrates that TP/TF drainage is a feasible and effective option for multiple right‐lobe bilomas, particularly when endoscopic ultrasound‐guided transmural drainage would require multiple transmural procedures, allowing endoscopic treatment to be completed without percutaneous drainage.

## 1 Introduction

Postoperative bile leaks are serious complications following hepatectomy, and localized biliary collections (bilomas) may become refractory to treatment. Bile leaks after elective hepatic surgery have historically been associated with substantial morbidity and mortality [1], making early intervention essential. Although endoscopic retrograde cholangiopancreatography (ERCP)‐based transpapillary drainage is the first‐line approach, it may fail in selected cases, including those with high‐grade leaks, intrahepatic ductal origin, or anatomical alterations after repeated hepatobiliary surgery [2]. Endoscopic ultrasound (EUS)‐guided transmural drainage (EUS‐TD) has also been reported as an alternative [3]. Although EUS‐guided drainage of right‐lobe collections is feasible [4], it is generally more technically demanding than for left‐lobe lesions. Moreover, when multiple bilomas are present, EUS‐TD requires individual transmural drainage for each lesion, imposing a substantial procedural burden.

In this context, transpapillary/transfistular (TP/TF) drainage, which allows direct access to the biloma cavity through the biliary fistulous tract, may be a rational option in cases with clear biliary communication [5]. However, reports on TP/TF drainage are scarce, and simultaneous TP/TF drainage for multiple right‐lobe bilomas has rarely been described. We herein report a case of two right‐lobe postoperative bilomas treated with TP/TF drainage, achieving stent‐free resolution over a 21‐month follow‐up, without the need for percutaneous drainage.

## 2 Case Report

A 57‐year‐old man with a history of type 2 diabetes presented with multiple hepatic metastases from rectal cancer. He had undergone robot‐assisted low anterior resection with ileostomy, followed by two sessions of laparoscopic partial hepatectomy with radiofrequency ablation and cholecystectomy for multiple metastases and residual liver recurrence. For further intrahepatic recurrence, partial hepatectomy of segments 7 and 6 with ileostomy closure was performed. No surgical drains were placed postoperatively.

**FIGURE 1:**
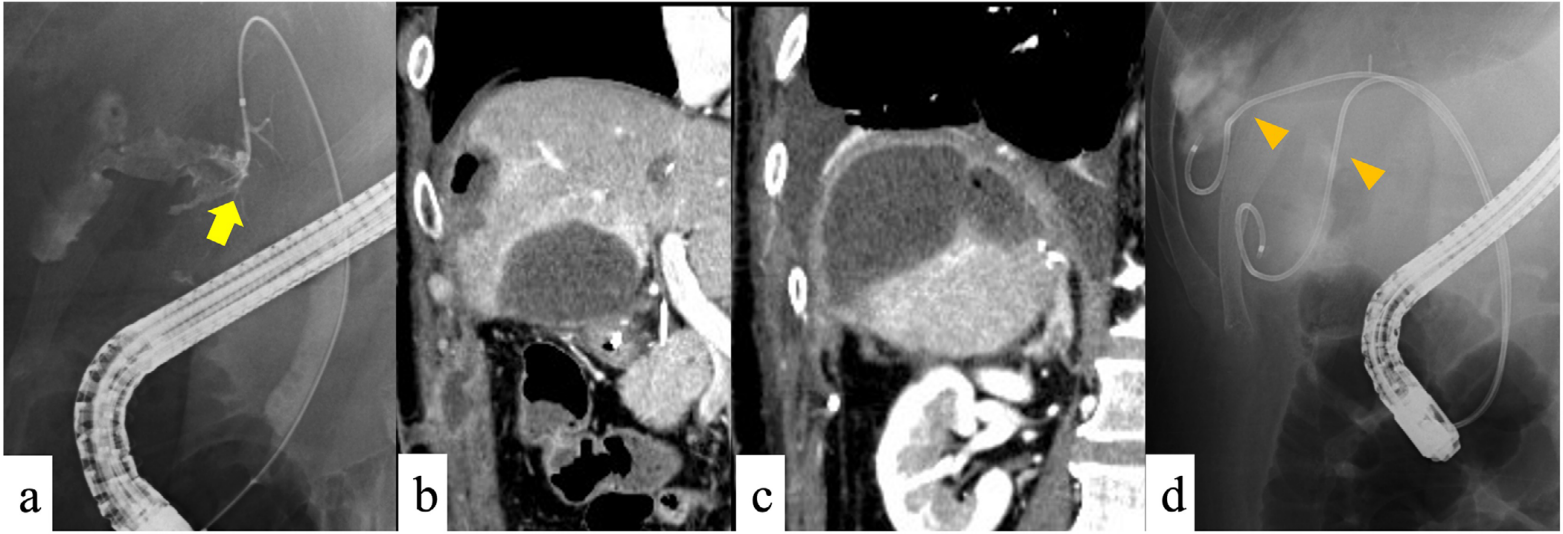
Initial endoscopic retrograde cholangiopancreatography (ERCP) findings and biloma enlargement after endoscopic nasobiliary drainage (ENBD) placement, and transpapillary/transfistular (TP/TF) drainage. (a) Fluoroscopic image during initial ERCP on postoperative day (POD) 6: the arrow indicates high‐grade bile leakage from B6, visible before complete intrahepatic opacification. (b) Coronal computed tomography (CT) on POD 11: an enlarged biloma (93 mm) in hepatic segment 6 (B6) after ENBD placement. (c) CT on POD 11: an enlarged biloma (71 mm) in segment 7 (B7). (d) Fluoroscopic image during TP/TF drainage on POD 14: two 5‐Fr pigtail‐type nasobiliary drainage tubes were advanced through the leak sites (arrowheads) and placed within the respective biloma cavities.

Computed tomography (CT) on postoperative day (POD) 6 revealed two bilomas measuring 68 mm in segment 6 (B6) and 60 mm in segment 7 (B7). The patient presented with fever (37.5°C) and elevated inflammatory markers (white blood cell [WBC] count 5490/µL and C‐reactive protein [CRP] 13.7 mg/dL), indicating the need for intervention (Clavien‐Dindo grade IIIa) [6]. ERCP was performed on POD 6, identifying high‐grade leaks from both B6 and B7 (extravasation before intrahepatic opacification; low‐grade leaks appear only after complete intrahepatic opacification) [7] (Figure 1a); a single endoscopic nasobiliary drainage (ENBD) tube was placed at the B6/B7 confluence; it drained bile and remained patent but did not bridge either leak site. However, repeat CT on POD 11 revealed enlargement of the bilomas to 93 mm (B6) and 71 mm (B7; Figure 1b,c).

Although inflammatory markers had improved (WBC 3,620/µL, CRP 5.0 mg/dL), fever persisted (38.5°C), and both bilomas had enlarged. TP/TF drainage was therefore performed on POD 14. Contrast‐enhanced CT before the procedure showed a well‐defined wall around each collection, confirming encapsulation. ERCP was performed using a side‐viewing duodenoscope (TJF‐Q290V; Olympus, Tokyo, Japan). After endoscopic sphincterotomy and biliary cannulation, a 0.025‐inch guidewire (VisiGlide; Olympus) was advanced through the bile leak sites into each biloma cavity. Balloon dilation of the fistulous tracts was performed using a 3‐mm balloon catheter (REN Type‐IT biliary balloon catheter; Kaneka Medix, Osaka, Japan), as the nasobiliary (NB) catheter could not be advanced without dilation. Following dilation, 5‐Fr pigtail‐type NB drainage tubes (NB‐Braid; Piolax Medical Devices, Kanagawa, Japan) were successfully placed within each biloma cavity (Figure 1d). No procedure‐related adverse events occurred.

**FIGURE 2:**
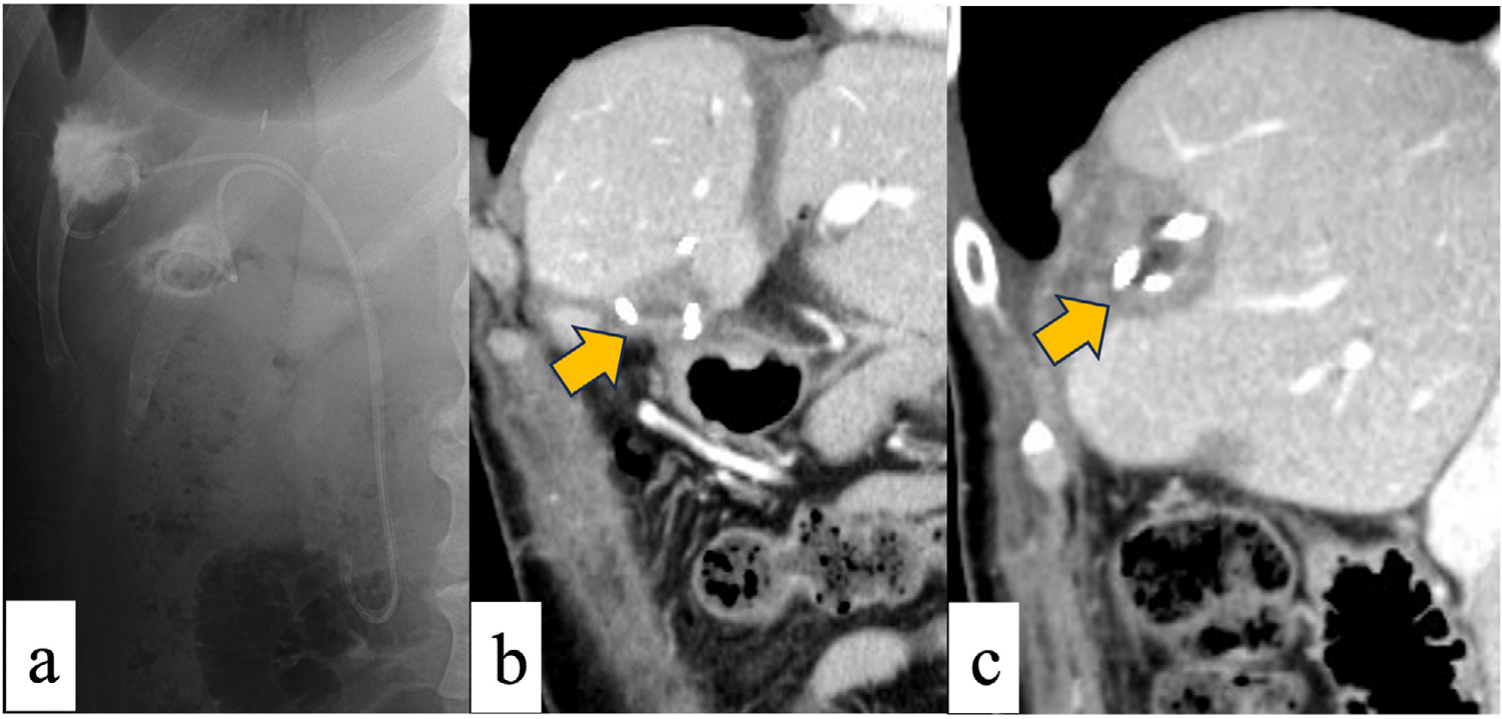
Internalization and biloma regression. (a) Fluoroscopic image during internalization on postoperative day (POD) 28: plastic stents (IYO‐stent, 5 Fr, 32 cm) were placed within each biloma cavity. (b) Coronal computed tomography (CT) after internalization: arrow indicates the markedly regressed B6 biloma. (c) CT after internalization: arrow indicates the regressed B7 biloma.

**FIGURE 3:**
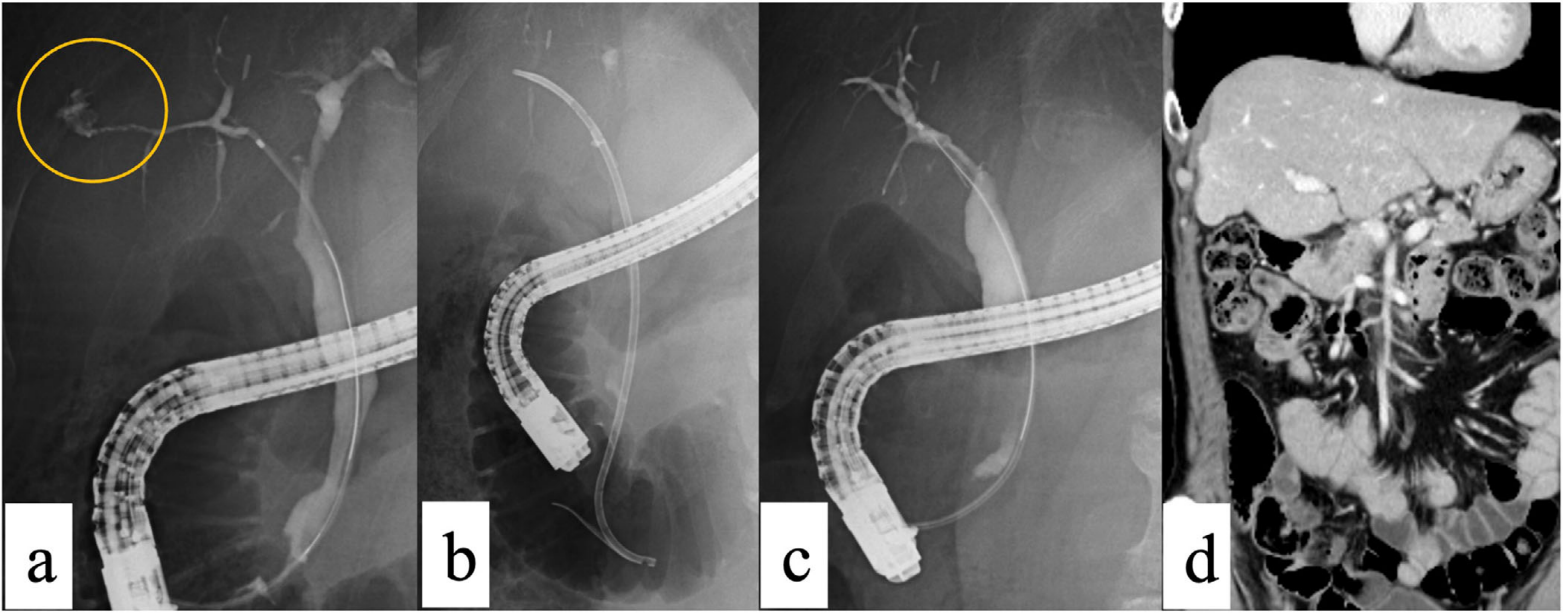
Additional internalization and treatment completion. (a) Fluoroscopic image at endoscopic retrograde cholangiopancreatography (ERCP) approximately 3 months after transpapillary/transfistular (TP/TF): the circle indicates low‐grade leakage from B7, representing improvement from the initial high‐grade leak. (b) A 7‐Fr plastic stent was placed in the B7 bile duct to complete internalization. (c) ERCP approximately 8 months after TP/TF: cholangiography revealed no biliary leakage, and the stent was removed. (d) Final computed tomography (CT) after stent removal: complete resolution of both bilomas was confirmed.

CT on POD 22 confirmed regression of both bilomas to 38 mm (B6) and 28 mm (B7). By POD 28, fever had resolved, inflammatory markers had improved (WBC 3,510/µL, CRP 3.1 mg/dL), and NB drainage output had become minimal. Given these findings, internalization was performed on POD 28. To place two guidewires simultaneously, two NB catheters were preloaded into the working channel of the duodenoscope prior to insertion. A 0.035‐inch guidewire (SeekMaster Hard; Piolax Medical Devices) was advanced through each NB catheter into the corresponding biloma cavity, and the NB catheters were then withdrawn, leaving both guidewires in place. After cholangiography confirmed the position of the guidewires within each biloma cavity, a plastic stent (IYO‐stent, 5 Fr, 32 cm; Gadelius Medical K.K., Tokyo, Japan) was placed over each guidewire into the respective cavity (Figure 2a); subsequent CT confirmed further regression (Figure 2b,c). ERCP approximately 3 months later demonstrated that the leak had improved to a low‐grade leak from B7 (Figure 3a). The drainage approach was therefore modified: a 7‐Fr plastic stent was placed in the B7 bile duct to complete internalization (Figure 3b). ERCP approximately 8 months after TP/TF drainage demonstrated no biliary leakage, and the stent was removed (Figure 3c); subsequent CT confirmed complete resolution of both bilomas (Figure 3d). The patient has remained stent‐free for 13 months without recurrence, with a total follow‐up of 21 months.

## 3 Discussion

In this case, initial ENBD via ERCP failed to control the bilomas, both of which enlarged after ENBD placement. High‐grade bile leak is an independent predictor of ERCP failure [8], and bilomas after hepatectomy are considered particularly challenging to manage endoscopically due to anatomical alterations [9]. In addition, the two bilomas were located in the right hepatic lobe. Although EUS‐guided drainage of right‐lobe collections is feasible in selected cases [4], it is technically more demanding than for left‐lobe lesions, and in the present case two separate transmural drainage procedures would have been required. We therefore did not perform EUS evaluation and instead selected TP/TF drainage, which allowed both bilomas to be addressed in a single session through their clear biliary communication.

A key advantage of TP/TF drainage is that it allows endoscopic treatment to be completed without percutaneous drainage. Lorenzo et al. [5] reported TP/TF drainage in a cohort that frequently included concomitant percutaneous drainage, leaving uncertainty as to whether endoscopic treatment alone is sufficient. In the present case, simultaneous TP/TF drainage of two biloma cavities allowed complete endoscopic management without any additional percutaneous intervention. This approach avoids external drain management and the bleeding and infection risks associated with percutaneous access. Simultaneous placement of two 5‐Fr NB tubes demonstrates the technical feasibility of TP/TF drainage in this anatomically challenging right‐lobe setting. Nevertheless, this strategy required repeated ERCP sessions, each carrying risks including post‐ERCP pancreatitis, bleeding, cholangitis, and perforation; although none occurred in this case, this cumulative burden should be weighed against the benefit of avoiding percutaneous drainage. Following internalization, the leak had improved to a low‐grade leak from B7, prompting a change in drainage strategy to plastic stent placement in the B7 bile duct, and complete resolution was ultimately achieved without serious adverse events. Conversely, for left‐lobe bilomas, EUS‐TD may be the preferred approach given the more favorable access from the stomach. A potential advantage of TP/TF drainage over EUS‐TD is that it avoids creating a transmural fistula, thereby reducing the theoretical risk of bile peritonitis.

The timing of internalization is a critical determinant of safety in TP/TF drainage. In percutaneous abdominal abscess drainage, residual collection volume below 50% of the initial volume has been identified as a predictor of favorable drainage outcomes [10]. Applying this principle to biloma management, we confirmed biloma regression to 38 mm (B6) and 28 mm (B7) on CT before proceeding with internalization on POD 28. Premature internalization before adequate biloma reduction risks exposing inflamed surrounding tissue and vulnerable vessels to stent‐related injury. Procedural safety also warrants comment. Balloon dilation was required in this case only because the NB catheter could not otherwise be advanced. Intentional dilation of an early postoperative bile duct disruption may theoretically aggravate bile extravasation or injure adjacent parenchyma or vessels, and should be reserved for cases in which device passage fails rather than performed routinely.

In conclusion, TP/TF drainage enabled complete endoscopic treatment of two refractory postoperative right‐lobe bilomas without percutaneous drainage. For multiple right‐lobe bilomas with clear biliary communication, TP/TF drainage may be a reasonable option, particularly when EUS‐TD would require multiple transmural procedures.

## Author Contributions

Fumitaka Niiya designed the study and wrote the manuscript. Naoki Tamai, Jun Noda, Tetsushi Azami, Yuichi Takano, and Masatsugu Nagahama collected data and revised the manuscript. All authors approved the final manuscript.

## Funding

The authors have nothing to report.

## Conflicts of Interest

The authors declare no conflicts of interest.

### Ethics Statement

This case report was approved by the Institutional Review Board of Showa Medical University Fujigaoka Hospital.

Written informed consent was obtained from the patient.
